# A 10-gene prognostic signature points to LIMCH1 and HLA-DQB1 as important players in aggressive cervical cancer disease

**DOI:** 10.1038/s41416-021-01305-0

**Published:** 2021-03-15

**Authors:** Mari K. Halle, Marte Sødal, David Forsse, Hilde Engerud, Kathrine Woie, Njål G. Lura, Kari S. Wagner-Larsen, Jone Trovik, Bjørn I. Bertelsen, Ingfrid S. Haldorsen, Akinyemi I. Ojesina, Camilla Krakstad

**Affiliations:** 1 Department of Obstetrics and Gynaecology, Haukeland University Hospital, Bergen, Norway; 2 Department of Clinical Science, Centre for Cancer Biomarkers, University of Bergen, Bergen, Norway; 3 Department of Clinical Medicine, University of Bergen, Bergen, Norway; 4 Department of Radiology, Mohn Medical Imaging and Visualization Centre, Haukeland University Hospital, Bergen, Norway; 5 Department of Pathology, Haukeland University Hospital, Bergen, Norway; 6 Department of Epidemiology, University of Alabama at Birmingham, Birmingham, AL USA; 7 O’Neal Comprehensive Cancer Center, University of Alabama at Birmingham, Birmingham, AL USA; 8 HudsonAlpha Institute for Biotechnology, Huntsville, AL USA

**Keywords:** Prognostic markers, Gene expression, Cervical cancer

## Abstract

**Background:**

Advanced cervical cancer carries a particularly poor prognosis, and few treatment options exist. Identification of effective molecular markers is vital to improve the individualisation of treatment. We investigated transcriptional data from cervical carcinomas related to patient survival and recurrence to identify potential molecular drivers for aggressive disease.

**Methods:**

Primary tumour RNA-sequencing profiles from 20 patients with recurrence and 53 patients with cured disease were compared. Protein levels and prognostic impact for selected markers were identified by immunohistochemistry in a population-based patient cohort.

**Results:**

Comparison of tumours relative to recurrence status revealed 121 differentially expressed genes. From this gene set, a 10-gene signature with high prognostic significance (*p* = 0.001) was identified and validated in an independent patient cohort (*p* = 0.004). Protein levels of two signature genes, *HLA-DQB1* (*n* = 389) and *LIMCH1* (LIM and calponin homology domain 1) (*n* = 410), were independent predictors of survival (hazard ratio 2.50, *p* = 0.007 for *HLA-DQB1* and 3.19, *p* = 0.007 for *LIMCH1*) when adjusting for established prognostic markers. HLA-DQB1 protein expression associated with programmed death ligand 1 positivity (*p* < 0.001). In gene set enrichment analyses, HLA-DQB1*high* tumours associated with immune activation and response to interferon-γ (IFN-γ).

**Conclusions:**

This study revealed a 10-gene signature with high prognostic power in cervical cancer. HLA-DQB1 and LIMCH1 are potential biomarkers guiding cervical cancer treatment.

## Background

Despite effective screening programs, cervical cancer is still the fourth leading cancer type in the female population worldwide leading to a fatal outcome for >311,000 women annually.^1^ Most cancer-related deaths are linked to tumour metastasis or recurrence of disease after primary treatment. In cervical cancer, 90% of recurrences occur within 3 years of initial diagnosis, and <5% of these patients survive beyond 5 years.^2^ Clearly, treatment regimens targeting these aggressive cervical carcinomas are presently suboptimal. Current first-line treatment for patients with metastatic cervical cancer includes platinum-based chemotherapy or paclitaxel/topotecan^3^ with overall response rates from recent trials ranging from 6 to 36%.^4–6^ Combination of chemo-based therapy with bevacizumab have shown improved survival with response rates of ~50%.^7^

As most cervical carcinomas have a viral aetiology, which impairs the immune system, immunotherapy by using checkpoint inhibitors or other immune-activating agents, appear as promising strategies. Recently, the PD-1 checkpoint inhibitor pembrolizumab gained accelerated approval for treatment of patients with recurrent or metastatic cervical cancers expressing programmed death ligand 1 (PD-L1).^8^ PD-L1 is now established as a predictive marker for immunotherapy, although overall response rates are as low as 14.3% for PD-L1-positive cervical cancer patients.^8^ Other PD-1 inhibitors, such as Nivolumab, are currently being tested in clinical trials (ClinicalTrials.gov identifier: NCT02488759)^9^ and results are pending. However, responses to available treatments for patients suffering from metastatic or recurrent cervical cancer are infrequent and often short-lived. Thus, better markers with which to predict response to these novel treatments are strongly needed. In addition, identification of biomarkers that can be used to safely stratify patients according to risk profile is essential to target treatment towards patients that are likely to benefit, while sparing those who will not.^10,11^

A key emphasis in the development of targeted treatment strategies involves unravelling the genomic landscape of the disease. In 2017, The Cancer Genome Atlas (TCGA) project provided a detailed molecular characterisation of 228 primary cervical carcinomas.^12^ While this study detected novel molecular features, subgroups and targets, the focus was not detection of prognostic markers. However, in the wake of this, several recent studies have performed outcome-based transcriptional analyses and have identified prognostic signatures within the TCGA dataset.^13–15^ However, a robust prognostic signature should be validated in external datasets and should ideally provide information regarding treatment decisions. This may be achieved by external validation and by unravelling the biological function of specific genes within the signature.

In this study, we aimed to compare primary tumour RNA expression profiles from 53 patients with cured disease to 20 patients with recurrent disease and compare findings to the independent TCGA cohort. To further characterise recurrent tumours and to pursuit possible treatment strategies, we explored whether differences in gene expression profiles were reflected in protein levels in a larger population-based validation cohort.

## Methods

### Patient cohorts

#### The primary investigation cohorts

Formalin-fixed paraffin-embedded (FFPE) tissue from all cervical cancer patients diagnosed and treated at the Department of Obstetrics and Gynaecology at Haukeland University Hospital in Bergen (Norway) has been prospectively collected in a population-based study from 2001 until 2017 (*n* = 444). Haukeland University Hospital is a referral hospital for patients in Hordaland County in Western Norway, representing ~10% of the Norwegian population with similar patterns of incidence and prognosis as from whole of Norway (Cancer Registry of Norway, http://kreftregisteret.no). All included patients have Caucasian ancestry, except five patients with Asian, two patients with Latin-American and two patients with African descent. Recruited patients were extensively characterised for clinical and histopathological data from primary diagnosis and follow-up data. All patients were clinically staged according to the International Federation of Gynaecology and Obstetrics (FIGO) 2009 criteria. Histological type and grade, depth of invasion, inflammatory reaction and vascular space invasion were histopathologically assessed by an expert pathologist, as previously described.^16^ Magnetic resonance imaging of the pelvis was performed at primary diagnostic work-up in 264 patients and included T2-weighted sequences acquired in two orthogonal planes. These were used to measure maximum tumour diameter on the slice depicting the largest maximum tumour diameter. All magnetic resonance imaging examinations were read independently by three radiologists, and the median value for maximum tumour diameter for the three readers was used for further analyses. Recurrence-free survival (RFS) was calculated from the date of primary treatment until verified disease relapse or metastasis or end of follow-up and disease-specific survival (DSS) from the date of primary treatment until death caused by cervical cancer or end of follow-up.

RNA-sequencing data were available for 80 patients, as previously described.^17^ This cohort included 79 patients with FIGO stage I and II and one with FIGO stage IV. To ensure a homogeneous cohort with clinical relevance, one case with FIGO stage IV was removed. Six of the patients had <5 years of follow-up and were not assigned to any prognosis group. Within the remaining FIGO stage I and II cohort, 20 patients recurred or died from cervical cancer during follow-up and were included in the ‘recurrent’ group, while 53 of the patients had RFS or DSS >5 years and were included in the ‘non-recurrent’ group.

#### TCGA validation cohort

The cervical cancer TCGA cohort consisting of 304 patients was used as an external validation cohort. The primary investigation cohort consists of cases with FIGO ≤ II only, and to ensure comparability of results, we excluded the FIGO < II TCGA cases from the comparative analyses involving the validation cohort. In total, within the FIGO I/II validation cohort, 96 patients matched the criteria of RFS > 5 years (‘non-recurrent’ group) and ten patients had recurrence or death during follow-up (‘recurrent’ group) (Supplementary Table 1). Clinical data from all patients and Fragments Per Kilobase of transcript per Million mapped reads (or FPKM values) for all RNA-sequenced genes were downloaded from the CBIO TCGA data portal (https://www.cbioportal.org/). Due to incomplete follow-up on disease-specific death, overall survival was chosen as end point for all survival analyses within the TCGA cohort. Overall survival was calculated from the date of primary treatment until death.

#### Clinicopathological characteristics across patient cohorts

Distribution of age at diagnosis, clinical FIGO stage, histological type and metastatic lymph node status for the different cohorts is presented in Table 1. Compared to the population-based cohort with tissue available for immunohistochemistry (IHC) assessment, the primary investigation cohort was enriched for low FIGO stage, as expected (*p* = 0.03). The TCGA validation cohort was enriched for high age (*p* = 0.03), high FIGO stage (*p* < 0.001) and squamous cell carcinomas (*p* < 0.001) when compared to the population-based cohort. The clinicopathological features were compared between recurrent and non-recurrent tumours in the primary investigation and the validation cohorts to detect potential confounders. No significant difference in FIGO stage, histological type and histological grade and distribution of metastatic lymph nodes were detected between recurrent and non-recurrent patients (Supplementary Table 1).

**Table 1:** Clinicopathological characteristics for patients within the population-based patient cohort applied for IHC assessment compared to the primary investigation cohort and the validation cohort (TCGA).

Variable	Cohorts, *n* (%)				
	Population based (*n* = 444)^a^	Primary investigation (*n* = 79)^b^	*P* value^c^	Validation (TCGA) (*n* = 304)^c^	*P* value^d^
Age at diagnosis (median)			0.14		**0.03**
<44	213 (48)	45 (57)		121 (39)	
≥44	231 (52)	34 (43)		183 (60)	
FIGO-09 stage			**0.03**		<**0.001**
I	322 (73)	67 (84)		161 (54)	
II–IV	122 (27)	13 (16)		136 (46)	
Histologic subtype			0.17		<**0.001**
SCC	318 (72)	49 (61)		253 (83)	
AC	91 (20)	22 (28)		45 (15)	
Other histology	35 (8)	9 (11)		6 (2)	
Metastatic lymph node			0.28		
No	250 (69)	57 (79)			
Yes	46 (31)	15 (21)			

*IHC* immunohistochemistry, *FIGO* The Féderation Internationale de Gynécologie et d’Obstétrique, *TCGA* The Cancer Genome Atlas, *SCC* squamous cell carcinoma, *AC* adenocarcinoma.

Statistically significant *p* < 0.05 values are in bold.

^a^Missing data in population-based cohort: metastatic lymph node = 148.

^b^Missing data primary investigation cohort: age, *n* = 1 and metastatic lymph node, *n* = 8.

^c^Missing data in validation (TCGA) cohort: FIGO, *n* = 7. Metastatic lymph node status was not available.

^d^Pearson’s *χ*^2^ test.

### Creating a 121- and 10-gene prognostic signature

**Fig. 1:**
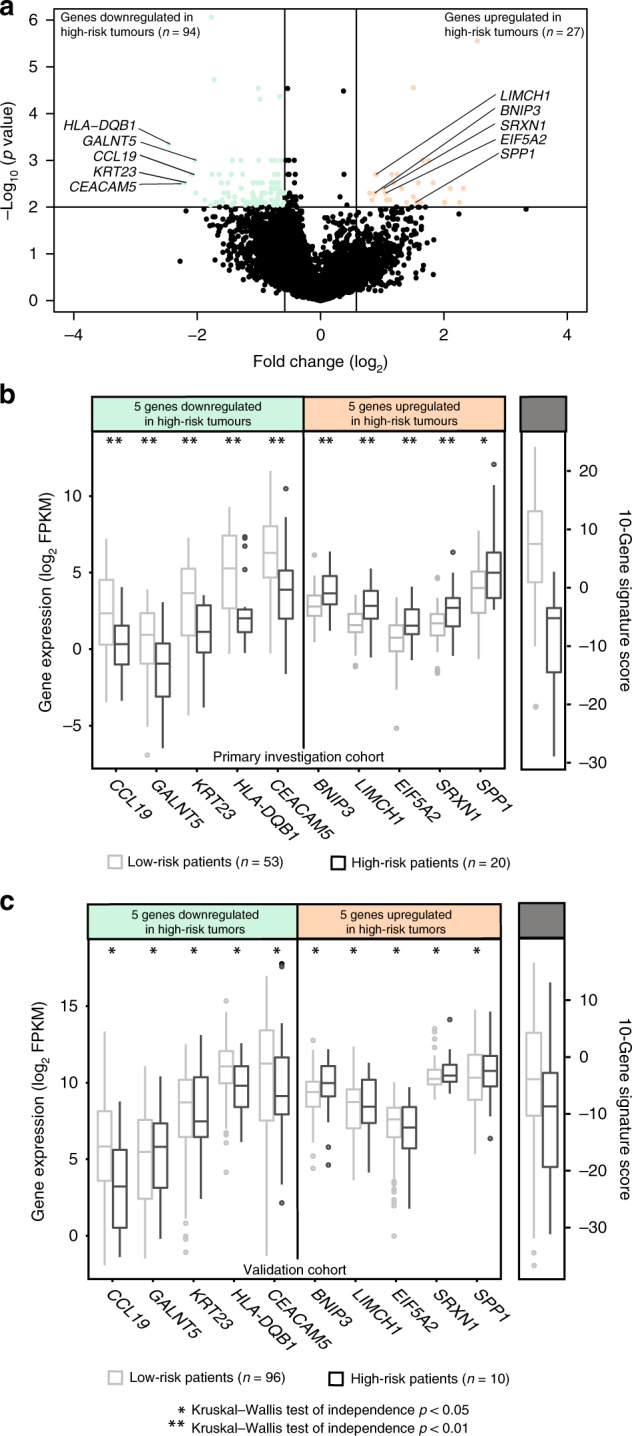
Identification of a prognostic signature. **a** Distribution of differentially expressed genes as defined by the criteria of *p* < 0.01 and fold change <−1.5 or >1.5. **b** Distribution of log 2 FPKM expression levels of the ten signature genes and the signature score in the primary investigation cohort and **c** the validation cohort relative to risk group. Expression values in the non-recurrent tumours are indicated by light coloured boxes and expression levels in recurrent tumours are indicated by dark coloured boxes. Symbols asterisk (*) and double asterisk (**) denote Kruskal–Wallis tests of independence of *p* < 0.05 and *p* < 0.01, respectively.

Feature subset selection (FSS) analyses were performed to identify differentially expressed genes (DEGs) between patients within the recurrent (*n* = 20) and non-recurrent (*n* = 53) groups. The 121-gene signature was created based on the significantly DEGs from the FSS matching the criteria of *p* < 0.01 and fold change <−1.5 or >1.5 and contained 27 genes that were upregulated and 94 genes that were downregulated in recurrent tumours. A signature score for each patient was created by subtracting the total expression value of 27 upregulated from the total expression value of the 94 downregulated genes. The signature was further reduced to only include ten genes by excluding transcripts with mean FPKM < 1. This FPKM threshold was chosen to maximise the probability of discovering biologically active transcripts and to minimise chances of detecting biological noise. Among the 27 upregulated genes, only *BNIP3*, *LIMCH1* (LIM and calponin homology domain 1), *EIF5A2, SRXN1* and *SPP1* (Fig. 1a) had FPKM > 1 and were selected for subsequent analysis. From the 94 downregulated genes, 70 genes had FPKM > 1 from which the five genes with highest fold change were selected for subsequent analysis: *CCL19*, *GALNT5*, *KRT23*, *HLA-DQB1* and *CEACAM5* (Fig. 1a).

### Tissue microarray (TMA)

FFPE tissue with corresponding haematoxylin- and eosin-stained full sections were collected from hospital archives for routine histopathological evaluation and IHC (*n* = 389, *n* = 410 and *n* = 434 for HLA-DQB1, LIMCH1 and PD-L1, respectively). For IHC, FFPE tissue was mounted in TMAs as previously described.^16^ The TMA method has previously been described and validated in several studies.^16,18–20^

The TMA sections were stained according to optimised IHC protocols for five selected antibodies (Supplementary Table 2) and were visualised and examined as previously described.^16^ The sections were assessed according to the staining index (SI), in which the combination of staining intensity (0–3) and affected cell area (0 = no staining, 1 = <10%, 2 = 10–50%, 3 = >50%) provided a subjective and semi-quantitative grading system. SI cut-off values defining high versus low protein expression for all antibodies are displayed in Supplementary Table 2. The intra-observer value of reproducibility within high versus low protein expression were 0.74, 0.61 and 0.85 for HLA-DQB1, LIMCH11 and PD-L1, respectively, when scored independently by two researchers (M.K.H. and M.S.). For PD-L1, all sections were also scored according to the combined positive score (CPS), which is the recommended method of evaluation for the Food and Drug Administration-approved commercial PD-L1 assays. The CPS is defined as percentage of positively stained neoplastic and mononuclear inflammatory cells. Less than 1% positive cells were defined as PD-L1-negative and ≥1% as PD-L1-positive tumours.

### Transcriptome analyses

DEGs were identified by using the FSS method within the JExpress software (www.molmine.com).^21^ The FSS ranking method was set to individual ranking to score the genes independently based on how they separated between groups (e.g., high versus low LIMCH1, HLA-DQB1 or PD-L1 protein expression). Gene set enrichment analyses (GSEAs) were performed within the JExpress software comparing tumours expressing high versus low LIMCH1, HLA-DQB1 and PD-L1, respectively (for cut-offs see Supplementary Table 2). Scoring method for GSEA was Golub (signal to noise) and permutations were performed on genes. C5, C6 and Hallmarks gene set collections of the Molecular Signature database v4.0 (MSigDB, Broad Institute, USA)^22^ were queried for enriched gene sets. A stromal and immune infiltration score was calculated for each patient with available RNA-sequencing data within the primary investigation cohort by using R version 3.6.3 (Massachusetts, USA) with the ESTIMATE (Estimation of Stromal and Immune cells in MAlignant tumour tissue using Expression) package version 1.0.13.^23^

### Statistical analyses

Statistical data analyses were performed using the Software package SPSS Statistics (Statistical Package of Social Science) version 25.0 (IBM, Armonk, USA). All probability values were two-sided and considered statistically significant if <0.05. Correlation between groups was assessed using Pearson’s *χ*^2^ or Fisher’s exact test as appropriate for categorical variables, while the Mann–Whitney *U* test was applied for continuous variables. Patient survival analysis was performed by applying the Kaplan–Meier (product–limit) method, and survival differences were determined by the log-rank test (Mantel–Cox). Receiver-operating characteristic (ROC) analyses were employed on the gene signatures to compare performance to predict risk group. Optimal gene signature cut-off values for dichotomisation applied in Kaplan–Meier analyses were identified from the ROC curves using the Youden index.^24^ Multivariate survival analyses were carried out using the Cox’s proportional regression hazard ratio (HR) method, adjusting for FIGO stage and age at primary diagnosis (for LIMCH1) or vascular space invasion (for HLA-DQB1) as appropriate according to known interactions between variables.

## Results

### A 10-gene signature identifies cervical cancer patients with poor survival

The clinicopathological characteristics of the different patient cohorts are displayed in (Table 1). Gene expression analyses within the primary investigation cohort identified 121 DEGs between non-recurrent (RFS and DSS > 5 years, *n* = 53) and recurrent (recurrence or death from disease, *n* = 20) tumours matching the criteria of *p* < 0.01 and fold change <−1.5 or >1.5. Of these genes, 27 were upregulated and 94 were downregulated in recurrent tumours (Fig. 1a and Supplementary Table 3). A signature score was calculated for each patient within the primary investigation cohort (for details see ‘Methods’ section) and a low signature score was strongly associated with poor survival (Supplementary Fig. 1A, HR = 59.5, *p* < 0.001). An analogous signature score for patients within the TCGA FIGO I/II validation cohort was calculated. Intriguingly, low signature score also predicted poor survival in the independent validation cohort (Supplementary Fig. 1b, HR = 2.64, *p* = 0.001). ROC curves for the 121-gene signature for prediction of risk group are displayed in Supplementary Fig. 1C, D, yielding area under the ROC curves of 0.95 and 0.82 for the primary investigation and the validation cohort, respectively.

**Fig. 2:**
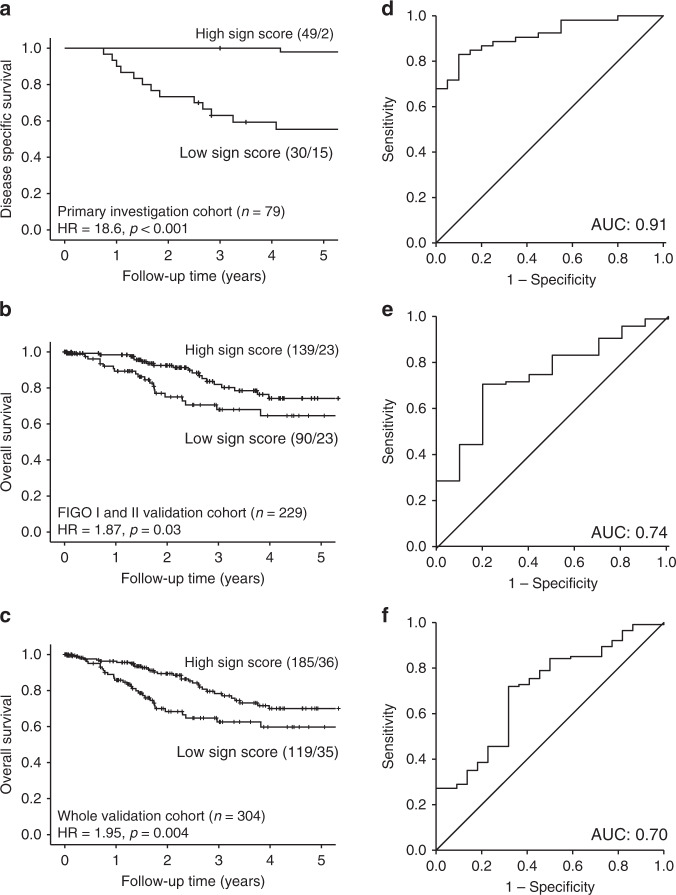
Prognostic impact of the 10-gene signature. **a** Disease-specific survival relative to signature score in the primary investigation cohort. **b** Overall survival relative to signature score in the FIGO I and II validation cohort. **c** Overall survival relative to signature score in the whole validation cohort. **a**–**c** Kaplan–Meier survival curves are presented with probability values for Mantel–Cox log-rank test that compares categories. The number of patients and events are given within parentheses (patients/events). **d**–**f** ROC curves are displayed to reflect the sensitivity and specificity of the gene signature to predict risk group in the primary investigation cohort (**d**), the FIGO I and II validation cohort (**e**) and the whole validation cohort (**f**).

To pinpoint key genes with possible prognostic impact and clinical utility, the signature was reduced to include ten genes (for details see ‘Methods’ section). Distribution of log 2 FPKM expression levels within recurrent and non-recurrent tumours for the ten signature genes and the signature score is displayed in Fig. 1b, c for the primary investigation and the validation cohort, respectively. Interestingly, reduction of the signature to include only these ten genes resulted in the maintenance of the correlation to survival in the primary investigation cohort (Fig. 2a, HR = 18.6, *p* < 0.001), the FIGO I/II validation cohort (Fig. 2b, HR = 1.87, *p* = 0.03) and the entire validation cohort (Fig. 2c, HR = 1.95, *p* = 0.004). ROC curves for the 10-gene signature to predict risk group are displayed in Fig. 2d–f with area under the curves of 0.91, 0.74 and 0.70 for the same three cohorts, respectively.

### LIMCH1 and HLA-DQB1 validate as prognostic markers for cervical cancer

**Fig. 3:**
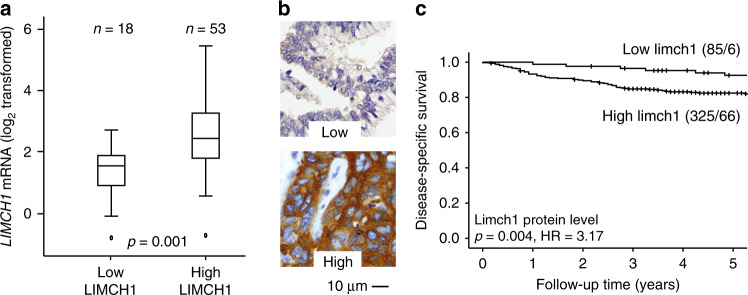
LIMCH1 protein levels relative to survival and mRNA expression. **a** mRNA levels of *LIMCH1* gene (log 2 transformed) relative to LIMCH1 protein expression levels in 71 tumours with overlapping transcriptional and immunohistochemical data. **b** Cancer tissue sections stained with LIMCH1 antibody with staining index (SI) score 0–3 (low) and 4–9 (high). **c** Disease-specific survival for cervical cancer patients relative to LIMCH1 protein expression levels represented by a Kaplan–Meier curve with probability values for Mantel–Cox log-rank test that compares categories. The number of patients and events are given within parentheses (patients/events).

Two genes within the 10-gene signature (*LIMCH1* and *HLA-DQB1*) were selected for further analyses as potential prognostic markers in a large population-based cervical cancer cohort by IHC performed in TMAs. High LIMCH1 protein levels were associated with higher *LIMCH1* messenger RNA (mRNA) levels (*n* = 71, *p* = 0.001) (Fig. 3a). LIMCH1 immunoreactivity was mainly cytoplasmic (Fig. 3b). Comparison of LIMCH1 expression with established clinicopathological markers revealed that LIMCH1*high* tumours were associated with non-squamous histological type (*p* = 0.05) and high tumour grade (*p* = 0.01) (Supplementary Table 4). In addition, high LIMCH1 expression was significantly associated with poor survival (*p* = 0.004, HR = 3.17) (Fig. 3c). In multivariate survival analysis, including FIGO stage and age at primary diagnosis, LIMCH1 protein expression independently predicted poor outcome, with adjusted HR of 3.19 (95% confidence interval (CI) 1.38–7.36, *p* = 0.007) (Supplementary Table 5). GSEAs revealed an enrichment of gene sets associated with ribosomal processes in LIMCH1*low* tumours (Supplementary Table 6).

**Fig. 4:**
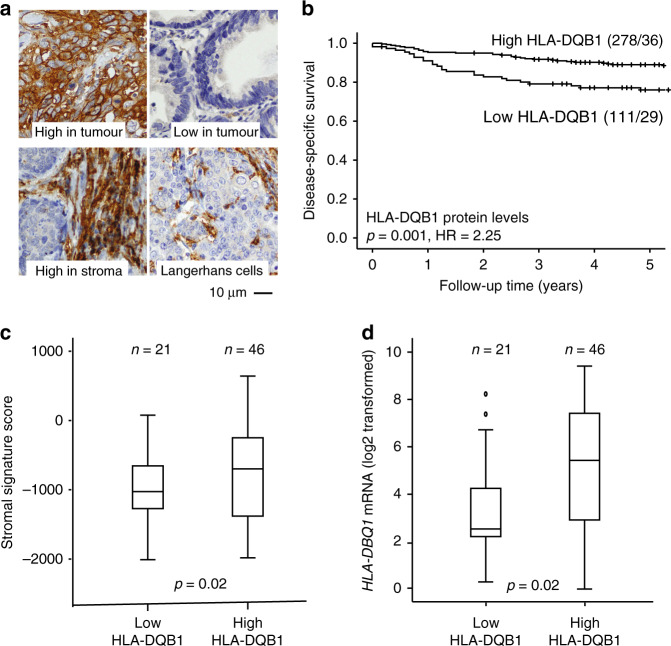
HLA-DQB1 protein levels relative to survival, stromal signature score and mRNA expression. **a** Cancer tissue sections stained with HLA-DQB1 antibody with staining index (SI) score 0–2 (low) and 3–9 (high). **b** Disease-specific survival for cervical cancer patients relative to HLA-DQB1 protein expression levels represented by Kaplan–Meier curves with probability values for Mantel–Cox log-rank test that compares categories. The number of patients and events are given within parentheses (patients/events). **c** Stromal signature score relative to HLA-DQB1 protein expression level in 67 tumours with overlapping transcriptional and immunohistochemical (tumour and stroma) data. **d** mRNA levels of *HLA-DQB1* gene (log 2 transformed) relative to HLA-DQB1 protein levels.

For HLA-DQB1, both tumour and stromal protein level were considered (*n* = 389, Fig. 4a). Of note, some tumours had scattered HLA-DQB1 staining patterns, with positive staining in Langerhans cells (Fig. 4a). HLA-DQB1*low* tumours were significantly associated with high FIGO stage (p < 0.001), tumour diameter >4 cm (*p* = 0.04), high histological grade (*p* = 0.03) and no or intermediate inflammatory reaction (*p* = 0.03) (Table 2). Further, HLA-DQB1*low* tumours were associated with poor DSS (Fig. 4b, *p* = 0.001, HR = 2.25); also when including FIGO stage and vascular space invasion in multivariate analyses (adjusted HR = 2.50; 95% CI: 1.29–4.87, *p* = 0.007, Supplementary Table 7). HLA-DQB1 expression was associated with a high ESTIMATE stromal cell infiltration score (*p* = 0.02) (Fig. 2c), and, interestingly, low stromal HLA-DQB1 levels associated strongly with poor survival (*p* = 0.003, HR 2.28, 95% CI 1.29–4.01, data not shown). HLA-DQB1 protein levels were significantly correlated with *HLA-DBQ1* mRNA levels (*p* = 0.02, Fig. 4d).

**Table 2:** HLA-DQB1 protein levels in tumour and stroma related to clinicopathological characteristics for 389 cervical carcinoma cases with evaluable results.

Variables (*n*)^a^	HLA-DQB1 protein levels		*P* value^b^
	Low (*n* = 111)	High (*n* = 278)	
Median age (*n* = 389)			0.07
≤44 years	45 (24)	141 (76)	
>44 years	66 (32)	137 (68)	
FIGO-09 stage (*n* = 389)			**<0.001**
I–IB1	57 (22)	196 (78)	
IB2–IV	54 (40)	82 (60)	
Max tumour diameter (*n* = 231)			**0.04**
<4 cm	30 (23)	100 (77)	
≥4 cm	36 (36)	65 (64)	
Histologic type (*n* = 389)			0.08
Squamous cell carcinoma	80 (29)	197 (71)	
Adenocarcinoma	19 (22)	66 (78)	
Other histologic type	12 (44)	15 (56)	
Histologic grade (*n* = 385)			**0.03**
Grade 1/2	86 (26)	239 (74)	
Grade 3	24 (40)	36 (60)	
Depth of invasion (*n* = 271)			0.44
Low (≤7 mm)	28 (21)	102 (79)	
High (>7 mm)	36 (26)	105 (74)	
Inflammatory reaction (*n* = 374)			**0.03**
No	18 (39)	28 (61)	
Intermediate	83 (29)	206 (71)	
Strong	5 (13)	34 (87)	
Vascular space invasion (*n* = 282)			0.51
No	45 (23)	148 (77)	
Yes	24 (27)	65 (73)	

The number of cases in each group is given followed by percentage for each row within parentheses.

*FIGO* The Féderation Internationale de Gynécologie et d’Obstétrique.

Statistically significant *p* < 0.05 values are in bold.

^a^*n* = number of cases with available data for each variable.

^b^Pearson’s *χ*^2^ test.

### High HLA-DQB1 protein levels indicate inflammatory reaction and immune activation

HLA-DQB1*high* tumours more often exhibited a strong inflammatory reaction based on histological full tumour sections (Table 2). Correspondingly, in GSEA analyses, the HLA-DQB1*high* tumours showed enrichment of gene sets related to inflammatory signalling pathways (e.g. IL6/JAK/STAT, TNFα/NFκβ and KRAS) and active immune response (e.g. immune response, T cell activation, granulocytes, complement system, cytokine signalling, leucocyte activation, interferon-γ signalling and inflammatory response) (Supplementary Table 8A, B). Within the GO gene sets, 78 out of the 100 top-ranked gene sets enriched in HLA-DQB1*high* tumours (false discovery rate < 0.0001) were associated with immune activation (Supplementary Table 8A).

To investigate whether patients with HLA-DQB1*high* tumours could be candidates for immune checkpoint inhibitors such as PD-1/PD-L1 inhibitors,^25^ we examined PD-L1 levels in 434 cervical carcinomas. Typical staining patterns for PD-L1 are shown in Supplementary Fig. 2A. No association was found between PD-L1 levels and prognosis, independently of the scoring method (*p* = 0.47 and *p* = 0.39 for SI and CPS, Supplementary Fig. 2B, C, respectively). For subsequent analyses, the SI scoring method was used to characterise patients with high versus low PD-L1. A significant association was found between PD-L1 expression and inflammatory reaction (p = 0.001) (Supplementary Table 9). PD-L1*high* tumours were significantly associated with HLA-DQB1*high* tumours (*p* < 0.001) (Supplementary Fig. 2D), and in total, 48% of the tumours (181/379) had collectively high PD-L1 and HLA-DQB1 levels. Furthermore, collectively high HLA-DQB1 and PD-L1 expression was significantly associated with high immune (*p* = 0.02) (Supplementary Fig. 2E) and stromal (*p* = 0.01) (Supplementary Fig. 2F) signature scores. When evaluating gene expression patterns in PD-L1*high* tumours by GSEA, only 24% of the significantly enriched GO gene sets (false discovery rate < 0.05) related to immune activation (Supplementary Table 10), indicating that HLA-DQB1 may be a stronger predictor for immune activation than PD-L1.

## Discussion

Despite large multicentre multi-omics efforts to characterise uterine cervical cancer, few targeted treatment strategies exist for patients suffering from metastatic or recurrent disease. In this study, we aimed to characterise the molecular profile of tumours from these patients with recurrence. To our knowledge, this is the first large-scale attempt to characterise specific molecular alterations distinguishing recurrent from non-recurrent cervical carcinomas by using two independent patient cohorts. We identified 121 DEGs between tumours from recurrent and non-recurrent patients. To increase the clinical applicability and pinpoint important features of aggressive disease, we reduced the signature to ten genes by eliminating genes that had mean FPKM values <1. The strong association to survival was observed within both the primary investigation cohort and the independent validation cohort (TCGA), indicating that this signature could be valid as a prognostic tool in different patient populations. We suggest that this signature should be tested in larger clinical trials to further optimise the prognostic power and clinical significance. Furthermore, five of the ten genes (*EIF5A2, SPP1, BNIP3*, *SRXN1* and *LIMCH1*) were upregulated in the recurrent tumours and could thus represent molecular drivers for aggressive cervical cancer disease.

Protein expression of LIMCH1 showed a strong association to poor prognosis. LIMCH1 positively regulates actin stress fibres assembly and stabilises focal adhesions through interaction with the actin-based motor protein non-muscle myosin II.^26,27^ LIMCH1 has been found to participate in the specific carcinogenesis of various types of cancer, including breast cancer, renal cancer and lung adenocarcinoma.^28–30^ In line with this, we found that LIMCH1*high* tumours associate with high *LIMCH1* gene levels, rare histological types, high grade and poor outcome in cervical cancer. Moreover, LIMCH1 expression demonstrated independent prognostic value when correcting for FIGO stage and age at primary diagnosis. In GSEA, 55% of the 20 top-ranked GO gene sets enriched in LIMCH1*low* tumours associated with ribosomal processes. This may suggest that LIMCH1 is involved in regulating translational processes in cervical cancer. LIMCH1 has previously been found to stabilise focal adhesions and accelerate cell contraction.^27^ Further, mRNAs and ribosomes have been found to localise to focal adhesions when cells bind to extracellular matrix-coated beads in an integrin- and actin cytoskeleton-dependent manner.^31,32^ This may suggest a regulative fuelling effect of ribosomes on the adhesion dynamics of cell migration. However, whether LIMCH1 plays a role in translation and whether this can be exploited in cervical cancer treatment needs to be determined.

We found *HLA-DQB1* to be the most upregulated gene within non-recurrent tumours and HLA-DQB1 protein levels associated significantly with favourable survival. HLA-DQB1 is a human leucocyte antigen (HLA) class II protein expressed by antigen-presenting cells. HLA class II proteins play a pivotal role in presenting foreign antigens to immune cells responsible for clearance of virus‐infected cells and tumour cells.^33^ We detected HLA-DQB1 expression in both tumour and stromal cells, which indicates that both tumour and infiltrating non-tumour cells may act as antigen presenters in cervical cancer. This is already known for several other cancer types,^34^ but to our knowledge not previously described in cervical cancer. In addition, we identified some tumours with more scattered staining patterns. Within these tumours, the highest staining intensity was typically detected in Langerhans cells, which are dendritic cells known as professional antigen-presenting cells in squamous cell carcinoma.^35^ This finding suggests that both professional (e.g. Langerhans cells) and non-professional (tumour cells) APCs present antigens via major histocompatibility complex (HLA) class II receptors in squamous cervical cancer.

The most potent stimulus for class II HLA proteins is IFN-γ, and when CD4^+^ T cells get activated by recognition of tumour antigens on HLA class II receptors, they produce additional IFN-γ, which subsequently induce further HLA class II expression and subsequent immune activation.^36^ Tumours surrounded by activated immune cells generally have a less aggressive phenotype than the immune suppressed tumours. Accordingly, we found that HLA-DQB1*high* expression associated with favourable DSS, activated immune response, high stromal infiltration score and histopathological inflammatory reaction. Conversely, HLA-DQB1*low* tumours may easily escape immune destruction and ultimately recur. In line with our findings, HLA-DQB1 expression has previously been found as a favourable prognostic marker in early-stage lung adenocarcinoma.^37^ Furthermore, we found an independent prognostic value of HLA-DQB1 after correction for FIGO stage and vascular space invasion, pointing to HLA-DQB1 as a possible prognostic marker in cervical cancer.

Almost all cervical cancers are HPV-driven, and virus-induced cancers are generally attractive targets for immunotherapy because viral proteins are strong immune stimulants.^38^ Indeed, immunotherapies targeting the PD-1/PD-L1 axis have provided long-lasting responses for some patients suffering from aggressive cervical cancer, yet the vast majority experience no clinical benefit. Pan-cancer clinical studies have revealed that apart from PD-L1 levels, mismatch repair deficiency, peripheral blood markers and high mutational and neoantigen load predict response to immune checkpoint inhibitors.^39–42^ These are all surrogate markers for tumours surrounded by active immune cells, or the so-called ‘hot’ tumours. To further investigate the potential of HLA-DQB1 as an indicator of immune activation, we investigated protein levels of HLA-DQB1 relative to PD-L1. We found a significant correlation between HLA-DQB1 and PD-L1 expression. More than 48% of the tumours had collectively high PD-L1 and HLA-DQB1 levels. These tumours associated significantly to high immune and stromal cell infiltration scores. Recently, Johnson et al.^43^ showed that HLA class II-positive tumours associated with CD4^+^ and CD8^+^ tumour infiltrate therapeutic response and improved survival in anti-PD-1-treated melanoma patients. In preclinical studies, they showed that HLA class II-positive tumours recruited the CD4^+^ T cells and developed dependency on PD-1.^44^ Further supportive of HLA-DQB1 as an important biomarker for immune activation in cervical cancer, our GSEAs suggest that HLA-DQB1 expression may be a stronger indicator of immune activation than PD-L1. Our findings combined with preclinical and clinical results in other cancer types suggest that the HLA class II receptor HLA-DQB1 may cause PD-1-dependent tumours in cervical cancer. Considering these findings, we suggest HLA-DQB1 as a potential marker for immune activation that may indicate a response to immunotherapy in cervical cancer. We propose that future clinical trials for PD-L1/PD-1 inhibitors also include HLA-DQB1 expression as an inclusion criterion.

We did not find any significant prognostic correlation between PD-L1 protein expression and DSS in our cohort of 434 cervical patients, independently of the scoring method. This is by far the largest single study evaluating the prognostic value of PD-L1 in cervical cancer, yet our findings are discordant to several previous studies. In 2019, Gu et al.^45^ performed a meta-analyses on 783 patients concluding that PD-L1 was significantly associated with poor outcome in cervical cancer (HR = 2.52, *p* = 0.03). Interestingly, in subgroup analysis based on ethnic descent, the link to survival was only maintained in patients of Asian descent. This may indicate that PD-L1 confers different associations to survival depending on ethnical origin.

Our transcriptional analyses are limited by the relatively small sample size within the primary investigation cohort, particularly the recurrent group, which includes 20 patients only. In general, a small sample size may camouflage significant biological features and may highlight coincidental associations. Furthermore, in the transcriptional analyses, FPKM values were applied. This limited us to identification of DEGs by the FSS method. Exploration of different algorithms to identify DEGs would thus be beneficial. However, strict cut-offs for DEGs were set and the signature was validated in an independent and large patient cohort. Moreover, protein and gene levels of selected signature genes showed high levels of concordance and a significant association to survival was detected at the protein level, confirming the prognostic value identified at the gene level. The primary investigation cohort reflects the Norwegian population, and 98% of the patients have Caucasian decent. In the validation (TCGA) cohort, 67% of the patients are Caucasian, 9% are Black or African American and 7% are Asian, reflecting mainly the American population. This difference in ethnical distribution, in addition to differences in stage at diagnosis due to, for example, higher screening rates in the Norwegian population, could explain differences found in age at diagnosis, stage and histology. Still, the signature was prognostic also within the validation cohort showing its relevance also within different ethnical and geographical populations.

In conclusion, this study reveals a 10-gene signature with high prognostic impact, also when assessed in an independent validation cohort. Two of the signature genes, *HLA-DQB1* and *LIMCH1*, displayed independent prognostic significance when investigated in a large population-based patient cohort by IHC, indicating a promising role as prognostic biomarkers guiding cervical cancer treatment. Furthermore, HLA-DQB1*high* tumours associated with inflammatory reaction, activated immune responses and PD-L1*high* levels pointing to HLA-DQB1 expression as a marker of immune activation in cervical cancer.

## Supplementary information

Supplementary Information

Supplementary Table 3

Supplementary Table 6

Supplementary Table 8

Supplementary Table 10

## Data Availability

The datasets used and/or analysed during the current study are available from the corresponding author on reasonable request.
