# Supplementary material for: A 10-gene prognostic signature points to LIMCH1 and HLA-DQB1 as important players in aggressive cervical cancer disease
Source: Br J Cancer. 2021 Mar 15;124(10):1690–8. doi: 10.1038/s41416-021-01305-0 (PMC8110544; doi:10.1038/s41416-021-01305-0)
Supplement: Supplementary file 1 — Supplementary Information [file 41416_2021_1305_MOESM1_ESM.docx]

**Supplementary Information**

**A 10-gene prognostic signature points to LIMCH1 and HLA-DQB1 as important players in aggressive cervical cancer disease**

Mari K. Halle^1,2*^, Marte Sødal^1,2^, David Forsse^1,2^, Hilde Engerud^1,2^, Kathrine Woie^1^, Njål G. Lura^3,4^, Kari S. Wagner-Larsen^3,4^, Jone Trovik^1,2^, Bjørn I. Bertelsen^5^, Ingfrid S. Haldorsen^3,4^, Akinyemi I. Ojesina^6,7,8^, and Camilla Krakstad^1,2^

*Supplementary Tables 3, 6, 8 and 10 can be found as separate excel files.*

**Supplementary Figure 1**


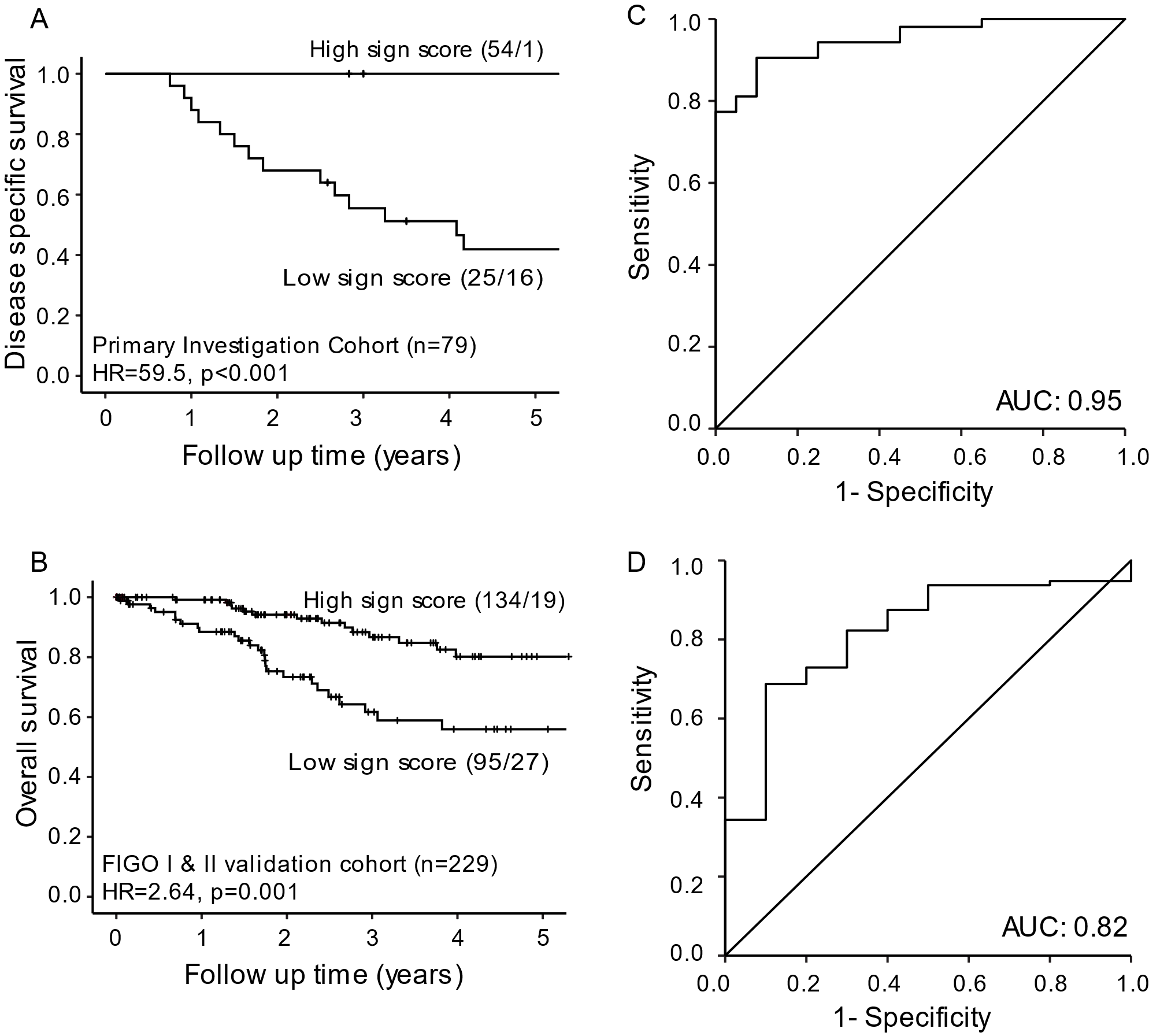


**Supplementary Figure 1: Prognostic impact of 121-gene signature**. **A)** Disease-specific survival relative to signature score in the primary investigation cohort represented in Kaplan-Meier survival curves. **B)** ROC curve reflecting the sensitivity and specificity of the 121-gene signature in the primary investigation cohort. **C)** Overall survival relative to signature score in the validation cohort represented in Kaplan-Meier survival curves. **D)** ROC curve reflecting the sensitivity and specificity of the 121-gene signature in the validation cohort. **A/C)** Kaplan-Meier curves are presented with probability values for Mantel-Cox log rank test that compares categories. The number of patients and events are given in parentheses (patients/events).

**Supplementary Figure 2**


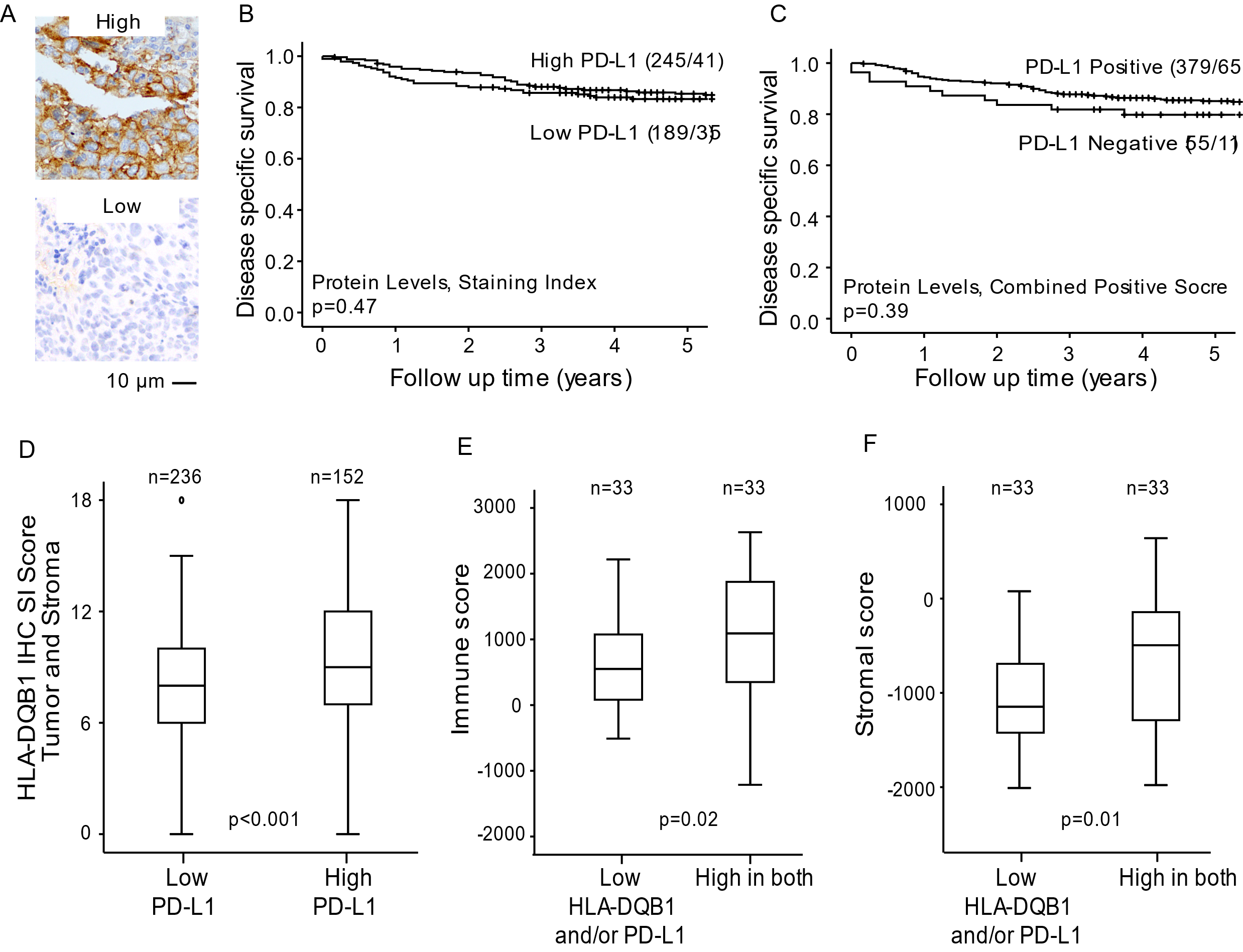


**Supplementary Figure 2: PD-L1 protein levels relative to survival, HLA-DQB1 protein levels and immune and stromal score. A)** Cancer tissue sections stained with PD-L1 antibody representing staining index (SI) score 0-2 (low) and 3-9 (high). **B & C)** Disease-specific survival for cervical cancer patients relative to PD-L1 SI score low *versus* high (**B**), and the Combined Positive Score (CPS) negative (<1%) *versus* positive (>1%) (**C**) represented by Kaplan-Meier curves with probability values for Mantel-Cox log rank tests that compare categories. The number of patients and events are given in parentheses (patients/events). **D)** Distribution of low *versus* high PD-L1 protein levels relative to HLA-DQB1 protein tumour and stroma levels. **E & F)** Immune (**E**) and stromal (**F**) score relative to PD-L1 and HLA-DQB1 protein levels.

**Supplementary Tables**

**Supplementary Table 1** Distribution of clinicopathological characteristics within risk groups in the primary investigation (n=73) and TCGA (n=106) FIGO II cohort with available RNA sequencing data. The number of cases in each group is given followed by percentage for each column in parenthesis.

|  | **Primary investigation cohort**  **n (%)** | | |  | **Validation cohort**  **n (%)** | | |
| --- | --- | --- | --- | --- | --- | --- | --- |
|  | ***Risk group n=73^a^*** | | |  | ***Risk group n=106^b^*** | | |
| ***Variables*** | ***Non-recurrent n=53*** | ***Recurrent***  ***n=20*** | ***P-value^b^*** |  | ***Non-recurrent***  ***n=96*** | ***Recurrent***  ***n=10*** | ***P-value^c^*** |
| *Median age in years* | *40* | *44* | *0.08^d^* |  | *46* | *45* | *0.74^d^* |
| *FIGO-09 stage* |  |  | 0.29 |  |  |  | 0.054 |
| IA | 0 (0) | 0 (0) |  |  | 1 (1) | 1 (10) |  |
| IB1 | 39 (74) | 11 (55) |  |  | 51 (53) | 2 (20) |  |
| IB2 | 8 (15) | 4 (20) |  |  | 10 (10) | 3 (30) |  |
| IIA | 4 (7) | 2 (10) |  |  | 15 (16) | 1 (10) |  |
| IIB | 2 (4) | 3 (15) |  |  | 19 (20) | 3 (30) |  |
| *Histologic type* |  |  | *0.13* |  |  |  | *0.64* |
| Squamous cell carcinoma | 32 (60) | 10 (50) |  |  | 81 (84) | 9 (90) |  |
| Adenocarcinoma | 17 (32) | 5 (25) |  |  | 15 (16) | 1 (10) |  |
| Other histologic type | 4 (8) | 5 (25) |  |  |  |  |  |
| *Histologic grade* |  |  | *0.19* |  |  |  | *0.67* |
| Grade 1/2 | 43 (83) | 13 (68) |  |  | 58 (65) | 4 (57) |  |
| Grade 3 | 9 (17) | 6 (32) |  |  | 31 (35) | 3 (43) |  |
| *Metastatic lymph nodes* |  |  | *0.23* |  |  |  |  |
| No | 42 (84) | 12 (71) |  |  |  |  |  |
| Yes | 8 (16) | 5 (29) |  |  |  |  |  |
| Abbreviations: FIGO: The Féderation Internationale de Gynécologie et d’Obstétrique  a: Primary investigation cohort: Patients without recurrence but with less than 5 years recurrence free survival (RFS) were excluded from the non-recurrent group (n=6). Missing data for histological grade: n=2 and metastatic lymph node: n=6  b: TCGA Cohort: Patients without recurrence but with less than 5 years RFS were excluded from the non-recurrent group (n=188). Missing data for age: n=1 and histologic grade: n=10. Metastatic lymph node status was not available for the validation cohort.  c: Pearson’s Chi-Square test  d: Independent Samples Mann-Whitney U Test | | | | | | |  |

**Supplementary Table 2:** Staining protocols, antibody provider and staining index cut-offs for clinicopathological and transcriptional analyses for HLA-DQB1, PD-L1 and LIMCH1.

| Protein targeted | Antigen retrieval | Primary antibody | Antibody dilution | Provider | Clinicopathological analyses* | Transcriptional analyses* |
| --- | --- | --- | --- | --- | --- | --- |
| HLA-DQB1 | Tris EDTA pH6 | Anti-HLA-DQB1 (HPA013667) | 1:75 | Sigma-Aldrich, Inc., Missouri, United States | SI low: 0-6 (n=111)  SI high: 7-18 (n=278) | SI low: 0-2 (n=13)  SI high: 6-9 (n=23) |
| PD-L1 | Tris EDTA ph6 | PD-L1 (E1L3N) Rabbit mAB | 1:100 | Cell Signaling Technology, Massachusetts, United States | SI low: 0-2 (n=189)  SI high: 3-9 (n=245)  CPS negative: <1% (n=55)  CPS positive: ≥1% (n=379) | SI low: 0-2 (n=53)  SI high: 3-9 (n=20) |
| LIMCH1 | Tris EDTA pH9 | Anti-LIMCH1 (HPA063840) | 1:500 | Sigma-Aldrich, Inc., Missouri, United States | SI low: 0-3 (n=85)  SI high: 4-9 (n=325) | SI low:0-3 (n=20)  SI high: 4-9 (n=53) |

*For the clinicopathological analyses of HLA-DQB1, histopathological staining of both tumour and stromal cells were considered and a combined staining index (SI) score for tumour plus stroma was applied. When PD-L1 was scored according to the Combined Positive Score (CPS), both tumour and immune cells were considered. For all other analyses only tumour cells were considered. For transcriptional analyses of HLA-DQB1 high *versus* low protein expression, tumours with intermediate staining (SI 3 or 4) were excluded.

Abbreviations: SI: Staining Index, CPS: Combined Positive Score.

**Supplementary Table 3 (*in separate excel file)*** List of 121 differentially expressed genes between high-risk (n=20) and low-risk (n=53) primary tumours included in the 121-gene signature. Log2 mean Fragments per Million Kilobase (FPKM) expression value for each gene was calculated across all investigated tumour samples in the primary investigation cohort. Genes with log2 FPKM value > 0 are highlighted.

**Supplementary Table 4** LIMCH1 tumour protein levels related to clinicopathological characteristics for 410 cervical carcinoma cases with evaluable results. The number of cases in each group is given followed by percentage for each row in parenthesis.

|  | ***Limch1 protein level*** | |  |
| --- | --- | --- | --- |
| ***Variables (n)^a^*** | ***Low***  ***n=85*** | ***High***  ***n=325*** | ***P-value^b^*** |
| *Median age (n=410)* |  |  | 0.36 |
| < 44 years | 45 (23) | 154 (77) |  |
| ≥ 44 years | 40 (19) | 171 (81) |  |
| *FIGO-09 stage (n=410)* |  |  | 0.60 |
| I-IB1 | 57 (22) | 208 (78) |  |
| IB2-IV | 28 (19) | 117 (81) |  |
| *Max tumour diameter (n=246)* |  |  | 0.80 |
| < 4 cm | 29 (21) | 112 (79) |  |
| ≥ 4 cm | 23 (22) | 82 (78) |  |
| *Histologic type (n=407)* |  |  | **0.047** |
| Squamous cell carcinoma | 68 (23) | 223 (77) |  |
| Adenocarcinoma | 15 (17) | 71 (83) |  |
| Other histologic type | 2 (6) | 31 (94) |  |
| *Histologic grade (n=406)* |  |  | **0.01** |
| Grade 1/2 | 79 (23) | 261 (77) |  |
| Grade 3 | 6 (9) | 60 (91) |  |
| *Depth of invasion (n=285)* |  |  | 0.77 |
| Low (< 7 mm) | 30 (22) | 106 (78) |  |
| High (≥ 7 mm) | 35 (23) | 114 (77) |  |
| *Inflammatory reaction (n=395)* |  |  | 1.00 |
| No/intermediate | 10 (21) | 38 (79) |  |
| Intermediate | 66 (21) | 243 (79) |  |
| Strong | 8 (21) | 30 (79) |  |
| *Vascular space invasion (n=297)* |  |  | 0.77 |
| No | 43 (21) | 161 (79) |  |
| Yes | 21 (23) | 72 (77) |  |

*FIGO*: The Féderation Internationale de Gynécologie et d’Obstétrique

^a^n=number of cases with available data for each variable

^b^Pearson’s Chi-Square test

**Supplementary Table 5** Survival analyses comparing FIGO stage and age at primary diagnosis with LIMCH1 protein expression according to the Cox’s proportional hazard regression method for 410 cervical cancer patients.

| Variables | Unadjusted HR | 95% CI | P-value | Adjusted HR | 95% CI | P-value |
| --- | --- | --- | --- | --- | --- | --- |
| FIGO stage ±IB1 (n=265 vs 145) | 6.05 | 3.61-10.1 | <0.001 | 5.38 | 3.18-9.10 | <0.001 |
| Age at primary diagnosis ±median  (n=199 vs 211) | 2.65 | 1.59-4.40 | <0.001 | 1.83 | 1.09-3.08 | 0.02 |
| LIMCH1 expression  Low *vs* high  (n=85 vs 325) | 3.17 | 1.37-7.31 | 0.007 | 3.19 | 1.38-7.36 | 0.007 |

Abbreviations: CI: Confidence Interval; FIGO: The Féderation Internationale de Gynécologie et d’Obstétrique.

**Supplementary Table 6** (*In separate excel file)* Top ranked gene sets from gene set enrichment analysis (GSEA) comparing low (n=20) versus high (n=53) LIMCH1 protein expression in cervical carcinomas using the C5 (GO) and C2 (curated) MSigDB gene sets.

Category indicates whether gene sets are related to ribosomal processes (1) or not (0).

**Supplementary Table 7** Survival analyses comparing FIGO stage and vascular space invasion with HLA-DQB1 protein expression according to the Cox’s proportional hazard regression method for 282 cervical cancer patients with available data.

| Variables | Unadjusted HR | 95% CI | P-value | Adjusted HR | 95% CI | P-value |
| --- | --- | --- | --- | --- | --- | --- |
| FIGO stage ±IB1  Low *vs* high  (n=224 *vs* 58) | 9.77 | 4.88-19.5 | <0.001 | 8.81 | 4.40-17.7 | <0.001 |
| Vascular space invasion  No *vs* Yes  (n=193 vs 89) | 2.90 | 1.50-5.60 | 0.002 | 2.88 | 1.48-5.59 | 0.002 |
| HLA-DQB1 expression  High vs Low  (n=69 vs 213) | 2.78 | 1.43-5.34 | 0.002 | 2.50 | 1.29-4.87 | 0.007 |

Abbreviations: CI: Confidence Interval; FIGO: The Féderation Internationale de Gynécologie et d’Obstétrique.

**Supplementary Table 8:** (*In separate excel file)* Top ranked gene cets from gene set enrichment analysis (GSEA) comparing high (SI6-9, n=23) *versus* low (SI 0-2, n=13) HLA-DQB1 protein expression in cervical carcinomas using the C5 (GO) (8A) and Hallmarks (8B) MSigDB gene sets.

Category indicates whether gene sets are related to immune activation (1) or not (0).

**Supplementary Table 9** PD-L1 tumour protein levels related to clinicopathological characteristics for 434 cervical carcinoma cases with evaluable results. The number of cases in each group is given followed by percentage for each row in parenthesis.

|  | ***PD-L1 protein level*** | |  |
| --- | --- | --- | --- |
| ***Variables (n)^a^*** | ***Low***  ***n=189*** | ***High***  ***n=245*** | ***P-value^b^*** |
| *Median age (n=434)* |  |  | 0.98 |
| ≤ 44 years | 90 (43) | 117 (57) |  |
| >44 years | 99 (43) | 128 (57) |  |
| *FIGO-09 stage (n=434)* |  |  | 0.76 |
| I-IB1 | 121 (43) | 160 (57) |  |
| IB2-IV | 68 (44) | 85 (56) |  |
| *Maximal tumour diameter (n=264)* |  |  | 0.38 |
| < 4 cm | 63 (42) | 87 (58) |  |
| ≥ 4 cm | 54 (47) | 60 (53) |  |
| *Histologic type (n=434)* |  |  | 0.48 |
| Squamous cell carcinoma | 130 (42) | 181 (58) |  |
| Adenocarcinoma | 43 (49) | 45 (51) |  |
| Other histologic type | 16 (46) | 19 (54) |  |
| *Histologic grade (n=430)* |  |  | 0.18 |
| Grade 1/2 | 162 (45) | 201 (56) |  |
| Grade 3 | 24 (36) | 43 (64) |  |
| *Depth of invasion (n=298)* |  |  | 0.54 |
| Low (≤ 7 mm) | 65 (43) | 85 (57) |  |
| High (> 7 mm) | 59 (40) | 89 (60) |  |
| *Inflammatory reaction (n=419)* |  |  | **0.001** |
| No | 34 (64) | 19 (36) |  |
| Intermediate | 141 (44) | 182 (56) |  |
| Strong | 11 (26) | 32 (74) |  |
| *Vascular space invasion (n=315)* |  |  | 0.14 |
| No | 102 (46) | 119 (54) |  |
| Yes | 35 (37) | 59 (63) |  |

*FIGO*: The Féderation Internationale de Gynécologie et d’Obstétrique

^a^n=number of cases with available data for each variable

^b^Pearson’s chi-square test

**Supplementary Table 10** (*In separate excel file)* Top ranked gene sets from gene set enrichment analyses (GSEA) comparing high (n=20) *versus* low (n=53) PD-L1 protein expression in cervical carcinomas using the C5 (GO) MSigDB gene sets.

Category indicates whether gene sets are related to immune activation (1) or not (0).
